# Lethal Means Counseling for Parents of Youth Seeking Emergency Care for Suicidality

**DOI:** 10.5811/westjem.2015.11.28590

**Published:** 2016-01-12

**Authors:** Carol W. Runyan, Amy Becker, Sara Brandspigel, Catherine Barber, Aimee Trudeau, Douglas Novins

**Affiliations:** * Colorado School of Public Health, Departments of Epidemiology and of Community and Behavioral Health, Program for Injury Prevention, Education and Research, Aurora, Colorado; † University of Colorado School of Medicine, Department of Psychiatry, Aurora, Colorado; ‡ Colorado School of Public Health, Program for Injury Prevention, Education and Research, Aurora, Colorado; § Harvard University, Harvard T. H. Chan School of Public Health, Boston, Massachusetts; || Colorado Department of Public Health, Denver, Colorado

## Abstract

**Introduction:**

A youth’s emergency department (ED) visit for suicidal behaviors or ideation provides an opportunity to counsel families about securing medications and firearms (i.e., lethal means counseling).

**Methods:**

In this quality improvement project drawing on the Counseling on Access to Lethal Means (CALM) model, we trained 16 psychiatric emergency clinicians to provide lethal means counseling with parents of patients under age 18 receiving care for suicidality and discharged home from a large children’s hospital. Through chart reviews and follow-up interviews of parents who received the counseling, we examined what parents recalled, their reactions to the counseling session, and actions taken after discharge.

**Results:**

Between March and July 2014, staff counseled 209 of the 236 (89%) parents of eligible patients. We conducted follow-up interviews with 114 parents, or 55% of those receiving the intervention; 48% of those eligible. Parents had favorable impressions of the counseling and good recall of the main messages. Among the parents contacted at follow up, 76% reported all medications in the home were locked as compared to fewer than 10% at the time of the visit. All who had indicated there were guns in the home at the time of the visit reported at follow up that all were currently locked, compared to 67% reporting this at the time of the visit.

**Conclusion:**

Though a small project in just one hospital, our findings demonstrate the feasibility of adding a counseling protocol to the discharge process within a pediatric psychiatric emergency service. Our positive findings suggest that further study, including a randomized control trial in more facilities, is warranted.

## INTRODUCTION

Suicide is the second leading cause of death in the United States for youth ages 12–17 years with mortality rates of 4.8 per 100,000 nationally in 2013 and a morbidity rate, as measured by emergency department (ED) visits for intentional self-harm, of 342.3 per 100,000. 1 Youth (ages 12–17) suicide attempts seen in emergency facilities are most often associated with poisoning, at a rate in 2013 of 151.5 per 100,000 2 while nearly half of completed suicides in this age group are associated with firearms. 3

One approach to addressing the risk of youth suicide is counseling families to reduce access to lethal means of self-harm (i.e., lethal means counseling). 4 Studies of lethal means counseling have demonstrated the potential effectiveness of this approach, 5–7 including one study that found parents made changes in storage of medications and guns after the counseling. 8 However, surveys of ED personnel and retrospective chart reviews indicate that many do not routinely engage in lethal means counseling with suicidal patients, suggesting that the dissemination and implementation of this intervention warrants more concerted attention. 9, 10

Our team evaluated a protocol aimed at improving the quality of lethal means counseling for parents of pediatric patients being discharged from the Children’s Hospital Colorado ED after a psychiatric assessment that addressed concerns about suicidal ideation or behavior. This counseling delivered by ED personnel was designed to: a) educate parents about common risks for suicide; b) urge parents to lock all medications in a lock box or other secure location; and c) encourage parents to store firearms away from the home or lock them securely during the mental health crisis period. 11

## METHODS

### Setting

The Psychiatric Emergency Service (PES) at Children’s Hospital Colorado provides round-the-clock access to specialty behavioral health providers (physicians and social workers) for pediatric patients coming for emergency care who are in behavioral health crisis. During the five-month project period (March 1, 2014 to July 31, 2014), the PES served over 1,405 children and adolescents.

### The Intervention

Our approach, modeled on the work of Kreusi 8 and Johnson, et al., 6 included a 1.5 hour online training for discharge counselors to support the delivery of a counseling session with families prior to discharge from PES and distribution of brochures and free lock boxes to these families. The online training, developed by Barber and Frank, 12 featured situations specific to adolescents seen in an ED, and guided counselors on how to ask and counsel families about safe storage of medications and firearms. The brochures (adapted from the Center to Prevent Youth Violence “Suicide-Proof” program) were in English and Spanish and included local resources for mental health services, medication disposal, and firearm storage. Families were offered free lock boxes ($59 value) suitable for storing medications. The adults accompanying the patients were informed that we would call them within several weeks to help us assess and improve our counseling procedures. They were not explicitly told that the call was to evaluate their compliance with recommendations.

The five-minute counseling session addressed the fact that access by the youth to medications and firearms was particularly dangerous and that parents should secure these items, at least during the child’s crisis. They were given suggestions on how to talk to the adolescent about suicidal feelings and were urged to temporarily remove guns from the home and lock medications where the teen could not access them.

All 14 behavioral health clinicians and two physicians who do discharge planning completed the training and implemented the intervention. The protocol also called for clinicians to complete a flow sheet through the electronic medical record, with drop-down menus prompting specific responses about the encounter. This included indications of the presence of unlocked medications and/or guns in the home, language preference, and whether brochures and lock boxes were given to the parent at discharge. The lethal means counseling was incorporated as part of the usual discharge process and did not disrupt normal patient flow.

We obtained approvals for this quality improvement project from the Children’s Hospital Colorado Organizational Research Risk and Quality Improvement Review Panel.

### Patient Population

PES clinicians counseled the accompanying adult (parent or guardian) of patients seen in the PES. Eligible patients were 12 to 17 years of age, endorsed suicidal ideation or had attempted suicide, and were being discharged home.

### Measures

We extracted information from the patients’ medical records and planned follow-up calls with the adults who accompanied the patient to the PES within two to three weeks post-discharge, confirming with them their phone numbers prior to discharge.

### Medical Record Data

Our data abstraction from medical records included a chart review to ascertain if all eligible patients seen during the time period were offered the intervention. We also extracted data from a patient flow sheet completed by the counselors to examine information recorded by providers about the presence of medications and guns in the home, how they were stored, whether the counseling was done (including provision of a brochure in English or Spanish), whether the family made any plans to change storage practices, and whether they accepted a lock box (with notation of the reason for refusals). The flow sheet also included contact information to enable follow-up interviews with the accompanying adults.

### Parent Interview

We trained three interviewers to conduct follow-up interviews using a computer-assisted survey via Survey Monkey. The protocol called for up to five calls to reach an adult family member who had received the lethal means counseling with the goal of completing the interview soon after being seen in the PES. For Spanish speakers, interviewers worked through the hospital translation service.

The telephone interview included a series of mostly close-ended questions about the parent’s recall of the storage status of medications and firearms in the home both at the time of the PES visit and the time of the interview, whether they recalled receiving discharge counseling, the most important messages they recalled, and their views about the counseling. All close-ended responses were recorded automatically in Survey Monkey requiring no further coding. We started with an open-ended question: “What was the most important thing you remember the counselor saying to you about medications in your home?” then continued with close-ended yes/no items that included: “Counselors talk about different things with different families based on their needs. Did your counselor talk about any of these things with you: a) Locking up prescription medications; b) Locking up all medications; c) Getting rid of old or expired medications.” To understand parent responses to the counseling, we asked a series of yes/no questions: “How did you feel about the way the counselor talked to you about the medications in your home--was it respectful of your family’s needs? “Were they clear about what they were recommending?” “Did they give you enough time to ask all your questions without you feeling rushed?” We had parallel questions specific to discussion of gun storage.

### Analyses

We compiled data in Microsoft Excel and conducted descriptive analyses of the rates of counseling among eligible families, details of the counseling process, and parent recall and assessment of the counseling. We computed chi-square or Fischer’s exact test statistics to assess differences between families counseled vs. not counseled and those interviewed vs. not interviewed.

## RESULTS

### Implementation of the Counseling

Of the 236 eligible families, 209 (89%) received the counseling intervention. Families counseled vs. not counseled did not vary significantly based on sex or age of the patient nor on whether the visit occurred during the day or night or on a weekday vs. weekend.

Clinician flow sheets indicated that they had discussed medication storage with 205 of the 209 (98%) counseled families. They discussed gun storage with 50 of the 52 (96%) of the families recorded as having guns in the home or 24% of 209 counseled families. Of the 236 eligible families, 79% accepted a free lock box and 86% were given brochures.

### Parent Participation in Interviews

Our interviewers were able to contact 55% (n=114) of the parents who accompanied a patient to the ED and whose records indicated they had received the counseling (see Figure). Among these, 104 interviews were conducted in English and 10 in Spanish. Seven were reached but refused to participate, and we could not make contact with 23 because of invalid phone numbers, missing names or numbers. Sixty-five parents did not answer repeated calls. We were not able to reach all within the planned 2–3 week period post-discharge but were able to complete all but five interviews within 90 days of the visit and all within 105 days.

To assess potential biases among our interview respondents, we compared the characteristics of the 114 counseled families who completed interviews with the 95 who received counseling but did not complete an interview. As shown in Table 1, parents who completed an interview did not differ significantly from those not interviewed on age, sex of patient, language preference, day of the week, time period of their visit, reported guns or medications at home, and acceptance of a lock box during the ED visit.

### Parent Response to Counseling and Materials

During the follow-up telephone interviews, 83 parents stated that they received the brochure, 13 indicated they did not receive it, and 18 could not recall. Among the 83 who reported receiving the brochure, 64 considered it “somewhat” or “very” helpful. However, 13 indicated they “didn’t know” or “didn’t read” it. Four reported it was not helpful and two did not respond.

Almost all (n=106) of those interviewed at follow up recalled the clinician talking about medication storage and indicated that the counselor was respectful of their family’s needs, clear about what they were recommending, and gave them enough time to ask questions. However, when asked if the counselor had discussed getting rid of old or expired medications, 51 said “yes,” 38 said “no” and 25 could not remember or didn’t respond.

We also asked respondents “What was the most important thing you remember the counselor saying to you about storing medications in your home?” More than 70 parents stated that they remembered being encouraged to lock up medications and/or to “keep them out of the reach of children” or in a “safe spot” such as in a locked room. Seven respondents indicated remembering that they should store all medications locked, while four mentioned over-the-counter medications and several focused on prescriptions. Five participants indicated they had been told to store knives in the lock box and two mentioned that they were also told to store cleaning supplies and chemicals in the lock box, though neither recommendation was intended as part of the intervention.

Thirty-three interviewed parents had indicated to the counselor at the time of their visit that they had guns in their homes, as recorded on the flow sheets. Among these, 26 (79%) recalled the counselor discussing with them how to store their guns. Most who remembered the discussion felt this part of the counseling was respectful of their family’s needs, was clear, and that the counselor allowed enough time for questions. Nineteen reported that the counselor had discussed temporarily storing guns out of the home, 26 indicated the counselor discussed storing guns in a locked cabinet or gun box, and 12 said the counselor talked about using trigger locks.

When asked what was the most important thing the counselor said about guns, 16 individuals said it was about locking the guns, with six also noting it should be kept unloaded as well and/or with ammunition stored separately. One gave a more generic answer (e.g., “not to have them within a child’s reach”). Eight specifically cited the discussion related to storing the firearm away from home during the mental health crisis.

### Parent Behaviors

Almost all (n=109) respondents who participated in the follow-up telephone interviews recalled having been offered a lock box for storing medications; 98 accepted it. Among those not taking the lock box, six indicated they already had one or locked their medications in a safe. Others gave varied reasons, ranging from not wanting to take one that might be needed by others less able to afford purchasing their own to being concerned that taking the lock box out of the hospital would have made their child self-conscious, while one stated that “[his/her child] will break into anything.”

At the time of the visit, flow sheet data indicated that 110 of the 114 parents later reached for follow-up interviews had reported to the counselor that they had medications in the home and 10 of these told the counselor that all of those medications were locked. When asked at the time of the follow-up interview about the current status of any unlocked medications in the home, 84 of 114 (76%, p=0.0016) reported that all medications in the home were locked (Table 2).

Counselors asked parents at the time of the visit about the presence of guns, with 52 families indicating they had guns in the home. Thirty-three of these 52 families participated in the follow-up interview (Table 2). Among these, 22 (67%) reported to the counselor at the time of the visit that their guns were all locked. At the time of the follow-up interview, all 33 parents (100%) reported that all of their guns currently were locked (p=0.0004).

## DISCUSSION

Most parents of children who met our eligibility criteria for counseling received lethal means counseling, demonstrating the feasibility of delivering the protocol in a pediatric emergency care setting. The vast majority of families were receptive to the discharge counseling and to receiving a free lock box during the visit. They described the discussions as being clear and respectful of their needs.

That parents showed good recall of key counseling messages at follow up, reported a high degree of acceptance of the intervention, and indicated substantial changes in storage practices is very encouraging and suggests the counseling was effective.

Though recall of key messages was strong overall, some remembered different messages than intended by the protocol (e.g., getting them out of the reach of children vs. locking them). Also, though the majority of families remembered being counseled about safe medication storage, the message regarding safe medication disposal was not consistently recalled. However, our quality improvement data does not allow us to determine if this reflects differences in how some clinicians may have delivered the intended messages or recall issues among the parents we interviewed. In some cases, more than one adult participated in the ED encounter while our interview was conducted with just one adult. It could be that different adults provided information to the clinician and to the interviewer, for example, reflecting variation in understanding of how guns or medications are stored in the home.

After the start of the project, the mental health team developed a checklist to remind clinicians of major points to include in their counseling. Larger-scale projects could examine in more detail if improvements are needed in the training protocol for clinicians; for example, giving stronger emphasis to consistency in providing the guidance to families, suggesting specific out-of-home gun storage locations to families; and using staff meetings to reinforce the importance of specific recommendations for storage and sharing ideas among personnel on how best to deliver those recommendations. Also, more developmental research to understand which messages are most persuasive with what types of patients could help guide this process.

As a group, self-reported storage practices changed substantially from the time of the visit to the ED and the time of the follow-up phone interview. These behavior changes are highly encouraging and suggest that giving adults a concrete way to effectuate safe storage (e.g., lock boxes) was helpful.

There are a number of cautions in considering the implications of our findings. First, this project took place in just one tertiary care facility with a relatively small number of clinicians and parents. Quality improvement projects like this one are designed to address service enhancement in a specific facility and are not intended to develop generalizable knowledge. This will require carefully designed research, ideally a randomized trial. Second, this facility has a 24/7 child and adolescent psychiatric emergency service, something that exists in a very small number of facilities. We cannot determine if our training and implementation model would have the same level of effectiveness in an ED with more limited access to behavioral health professionals specializing in the care of children. Third, the project design relied on self-report regarding counseling delivery and changes in storage practices. It is possible that, due to a social desirability concerns, some parents reported that they had locked up their medications and/or locked firearms or stored them away from home when in fact they had not. To mitigate social desirability influences, we made a point to ask behavior change questions at the start of the interview before questions about the counseling encounter itself. We cannot be sure this alleviated the potential problem. Finally, we completed follow-up interviews with just 55% of the recipients of the intervention, raising the risk of non-responders having had a different experience with this counseling. However, the fact that participants and non-participants did not differ on key variables is reassuring.

Provision of the free lock boxes was enabled by grant funding from a foundation. The feasibility of providing free or reduced-cost lock boxes will depend upon the ability of a given provider to identify a stable funding source for lock box purchase. Future research should seek to determine if providing free or low-cost lock boxes are a cost effective and critical part of the intervention.

In this intervention, we debated whether to counsel families to lock all medications or only the most lethal. It is unknown if it is advisable to counsel parents to lock only the more toxic drugs and leave small quantities of low-toxicity medications unlocked in case the child is inclined to substitute and attempt suicide with another method. The drawback is that such a shift in protocol would require more training, more complicated messaging to families, and potentially reduced compliance by parents because of the more complex task involved. Future research should examine this further.

A review of studies that examined repetition of self-harm found that on average 5–11% of people treated for an index suicide attempt go on to die by suicide, 13 a far higher suicide rate than in the general population. Access to a firearm in particular is a risk factor for completed suicide. 14–16 It is encouraging that among the nine suicidal youth whose families received the intervention and had had unlocked guns at home at the time of their ED visit, none reported unlocked guns at follow up. One outcome that was not explicitly assessed was whether parents took steps to store firearms away from home after the PES visit. This should be assessed in future work.

Expansion of the intervention may also be warranted. The protocol evaluated here was delivered only to adults accompanying patients who were being discharged home from the ED after evaluation by the PES team. We did not examine how counseling is done with the families of youth transferred to inpatient care prior to discharge and cannot generalize to facilities where care is delivered by providers without mental health credentials. We also did not differentiate responses among families whose child presented with suicidal ideation vs. an attempt to determine if response to the intervention differed. This will be important to examine in future research. The interval between the ED visit and an adolescent’s return home from inpatient care presents another potential opportunity for families to change their medication and firearm storage and is worthy of more careful investigation.

While more research is warranted, addressing lethal means counseling as a part of routine emergency care is increasingly being advocated as a critical element of provider training and practice. 17 This project suggests that lethal means counseling for parents of suicidal youth is feasible to incorporate into emergency care in a way that is acceptable to them and results in positive behavior change.

## Figures and Tables

**Figure:**
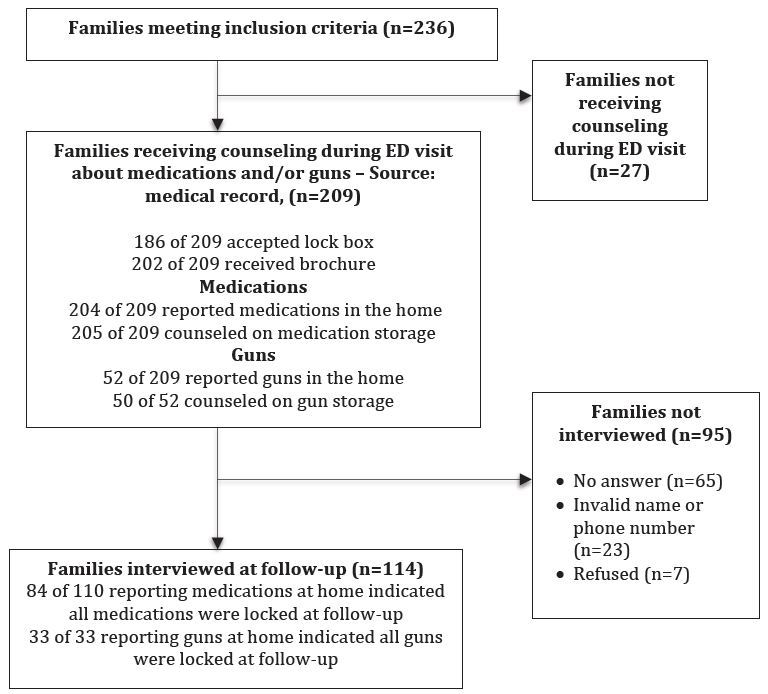
Receipt of counseling, participation in follow-up interviews, and family reports of storage behaviors. *ED,* emergency department

**Table 1:** Characteristics of families with completed follow-up interview (n=114) vs. no interview (n=95) based on data from medical record.

Characteristics (derived from medical record)	Interviewed (n=114)^B^ n (%)	Not interviewed (n=95)^B^ n (%)
Sex of patient (p=0.71)		
Female	82 (72%)	66 (69%)
Age of patient (p=0.17)		
<15	60 (53%)	59 (62%)
15+	54 (47%)	36 (38%)
Language preference (p=0.75)		
English	100 (88%)	80 (84%)
Spanish	13 (11%)	12 (13%)
Time of arrival to ED^A^ (p=0.8)		
Daytime (7AM–6:59PM)	68 (60%)	59 (62%)
Nighttime (7PM–6:59AM)	36 (32%)	30 (32%)
Day of arrival to ED^A^ (p=0.10)		
Weekday	90 (79%)	69 (73%)
Weekend	14 (12%)	20 (21%)
Reported guns at home at time of ED visit (p=0.14)	33 (29%)	19 (20%)
Reported medications at home at time of ED visit (p=0.38)	110 (96%)	94 (99%)
Accepted a lock box during visit (p=0.28)	99 (87%)	87 (92%)

*ED*, emergency department

A Based on time of triage recorded in medical record.

B Missing data for some variable for 16 patients.

**Table 2:** Comparison of medication and gun storage behavior reported by parents at time of visit and in follow-up interview among those completing a follow-up interview (n=114).

	At the time of ED visit n (%)^A^	At the time of follow-up n (%)^B^	Statistical test of change over time period
Medication storage among families indicating they had medications in the home at visit (n=110, 96%)			
All locked^C^	10 (9%)	84 (76%)	p=0.0016^E^
Gun storage at time of ED visit among families indicating they had guns in the home at visit (n=33, 29%)			
All locked^D^	22 (67%)	33 (100%)	p=0.0004^F^

*ED,* emergency department

A From flow sheet data recorded by counselor on day of visit.

B From follow-up interview.

C At the visit, we recorded 9 (8%) reporting some were locked; 89 (81%) reporting none were locked, and 2 (2%) responses were missing or reported “not sure”. At follow-up the question only asked if there were “any unlocked medications at home today”. Data missing for one family.

D At the visit, we recorded no families reporting some guns were locked, 9 (27%) reporting that none were locked and 2 (6%) indicating they were unsure of whether guns were locked or not. At follow-up, the question asked if there were “unlocked guns at home today”.

E Chi Square test.

F Fisher’s Exact test.
